# Annoyance from Road Traffic, Trains, Airplanes and from Total Environmental Noise Levels

**DOI:** 10.3390/ijerph13010090

**Published:** 2015-12-29

**Authors:** Martina S. Ragettli, Sophie Goudreau, Céline Plante, Stéphane Perron, Michel Fournier, Audrey Smargiassi

**Affiliations:** 1 Department of Environmental Health and Occupational Health, School of Public Health, University of Montreal, Montreal, QC H3C 3J7, Canada; Martina.Ragettli@umontreal.ca; 2 Public Health Department of Montreal, Montreal, QC H2L 1M3, Canada; sgoudrea@santepub-mtl.qc.ca (S.G.); cplante@santepub-mtl.qc.ca (C.P.); sperron@santepub-mtl.qc.ca (S.P.); mfournier@santepub-mtl.qc.ca (M.F.); 3 Swiss Tropical and Public Health Institute, Basel 4002, Switzerland; 4 University of Basel, Basel 4003, Switzerland; 5 National Institute of Public Health of Quebec, Montreal, QC H3C 2B9, Canada; 6 Public Health Research Institute, University of Montreal, QC H3C 3J7, Canada

**Keywords:** environmental noise, exposure, annoyance, transportation, Canada

## Abstract

There is a lack of studies assessing the exposure-response relationship between transportation noise and annoyance in North America. Our aims were to investigate the prevalence of noise annoyance induced by road traffic, trains and airplanes in relation to distance to transportation noise sources, and to total environmental noise levels in Montreal, Canada; annoyance was assessed as noise-induced disturbance. A telephone-based survey among 4336 persons aged >18 years was conducted. Exposure to total environmental noise (A-weighted outdoor noise levels—LA_eq24h_ and day-evening-night equivalent noise levels—L_den_) for each study participant was determined using a statistical noise model (land use regression—LUR) that is based on actual outdoor noise measurements. The proportion of the population annoyed by road traffic, airplane and train noise was 20.1%, 13.0% and 6.1%, respectively. As the distance to major roads, railways and the Montreal International Airport increased, the percentage of people disturbed and highly disturbed due to the corresponding traffic noise significantly decreased. When applying the statistical noise model we found a relationship between noise levels and disturbance from road traffic and total environmental noise, with Prevalence Proportion Ratios (PPR) for highly disturbed people of 1.10 (95% CI: 1.07–1.13) and 1.04 (1.02–1.06) per 1 dB(A) L_den_, respectively. Our study provides the first comprehensive information on the relationship between transportation noise levels and disturbance in a Canadian city. LUR models are still in development and further studies on transportation noise induced annoyance are consequently needed, especially for sources other than road traffic.

## 1. Introduction

Annoyance is the most prevalent health effect in a population exposed to environmental noise. It can result from noise disrupting people during daily activities, sleep or rest and may cause a variety of negative responses such as anger, distraction, depression, anxiety, exhaustion and stress-related symptoms [1,2]. Noise annoyance has further been shown to be associated with reduced quality of life [3,4] and well-being [2]. Traffic noise is one of the main sources of annoyance. Several studies have shown positive exposure-response relationships between annoyance and increasing environmental noise levels induced by road traffic, trains and airplane movements [4,5,6,7]. 

The exposure to environmental noise from transportation sources and its related health burden is of increasing concern. In urban areas, road traffic is the most widespread noise source. According to the World Health Organization (WHO) Europe, exposures to A-weighted day-evening-night equivalent sound pressure level (L_den_) of road traffic noise that exceed 55 dB(A) pose a serious risk to health [1,8]. In 2011, it was estimated that about 50% of the population in large agglomerations (>250,000 inhabitants) of the European Union (EU) was exposed to road traffic noise levels (L_den_) above 55 dB(A) [8]. 

While sensitivity and exposure to noise may differ between regions of the globe (e.g., due to differences in house construction, window opening habits), the relation between environmental noise levels and annoyance has mostly been studied in Europe [2,9]. For example, the EU has implemented the Environmental Noise Directive 2002/49/CE [10] to assess and reduce population-wide exposure to noise from transportation sources. Similar national initiatives in other countries in the world are rare. In Canada and the United Sates (US) for example, no standardized noise estimation approaches exists [11,12].

Furthermore, thus far, the relation between environmental noise levels and annoyance has only been established with modeled environmental noise levels that are not based on actual noise measurements but simulated with noise propagation models [4,5,8]. Such numerical models predict noise for a specific source by means of physical rules of noise propagation and attenuation [13]. Recent noise monitoring campaigns in the US [11], and in the three Canadian cities Halifax [14], Toronto [15] and Montreal [16], have shown that measured noise levels tend to exceed the 55 dB(A) threshold that has been associated with negative health impacts mainly in European studies using noise estimates from propagation noise models [1,8]. Furthermore, according to a national survey performed in Canada in 2005, 6.7% of Canadians reported being highly annoyed by road traffic noise, with generally higher percentages found in larger cities than in smaller communities [17]. In view of the ever-growing urban populations and public health concerns about excessive transportation noise in North American cities, studies investigating exposure-response relationships and potential noise-related health effects are warranted.

The current study presents data of a telephone-based survey on noise annoyance carried out in 2014 on the Montreal Island (Canada). Our aims were to investigate the prevalence of annoyance induced by road traffic, trains and airplanes in relation to (a) distance to transportation noise sources; and (b) to total environmental noise levels estimated with a land use regression model that is based on an extensive noise measurement campaign carried out in Montreal [18].

## 2. Methods 

### 2.1. Study Population

**Figure 1:**
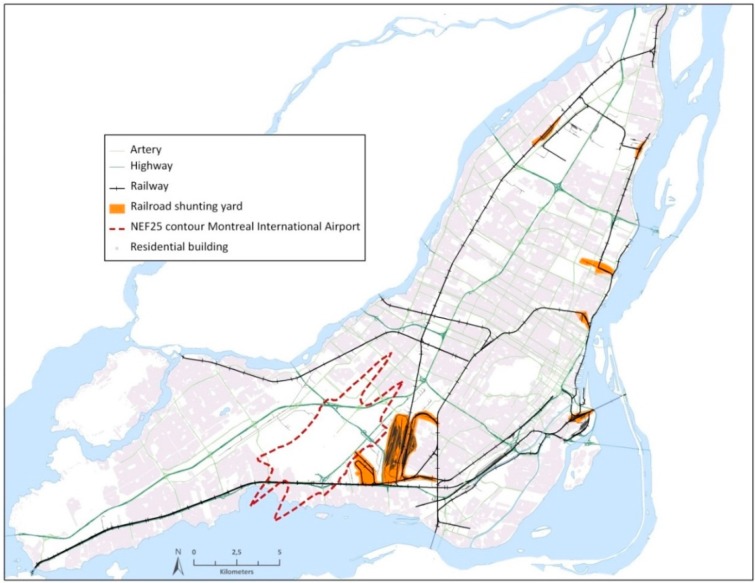
Map of the study area with potential transportation noise sources.

There were about 1.9 million people living on the Island of Montreal in 2011 [19], on an area of approximately 500 km^2^ (Figure 1). Montreal Island includes 14 municipalities among which belongs the city of Montreal—that includes most of the population of the Island—and which is the second largest city in Canada. A significant fraction of the population lives close to major roads such as arteries and national highways (e.g., Trans-Canada Highway). Some residences are also located in proximity to railway tracks and/or in the vicinity and in the flight paths of the Montreal International Airport. Hereafter, the zone around the airport is referred to as the Noise Exposure Forecast zone 25 (NEF25). Defined by the Airport Authority and used for urban planning purposes, it describes an area where annoyance is likely to occur in the surrounding of the airport [20]. 

To ensure a sufficient number of subjects exposed to each transportation noise source, survey respondents >18 years of age were randomly selected from pre-defined survey strata (*road, rail, NEF25, not-exposed*). To build these strata, the population on the island was first divided in seven transportation noise exposure categories by means of ArcGIS 9.2 (ESRI, Redlands, CA, USA) using a digital road network (Addresses Québec 2013), a railway network (CanMap^®^ Rail V2010.1), and the geo-referenced NEF25 map of the Montreal International Airport [16,21]. The exposure categories were defined based on the results of previous noise exposure studies in Montreal reporting noise levels in the vicinity of transportation noise sources [16,22,23] and to ensure sufficient numbers of respondents in each strata as follows: people living within 100 m of an artery or highway (road), within 150 m of a main railway line or main line of a railroad shunting yard (rail), within 1 km of the NEF25 zone of the Montreal International Airport (NEF25), as well as the combinations of the road, rail and NEF25 categories (*i.e.*, road and rail, road and NEF25, rail and NEF25, road and rail and NEF25). People living within those categories are considered to be most exposed to the respective noise sources. The categories were then combined to four survey strata based on their population share: (1) *road* only; (2) *rail* (rail and road and rail); (3) *NEF25* (NEF25, road and NEF25, rail and NEF25, road and rail and NEF25); and (4) *non-exposed* (an area not directly exposed to transportation noise sources). 

In each stratum, six digit residential postal codes were identified and linked to a local telephone number registry and then selected randomly for interviews. Each six-character postal code usually represents a street block or a large apartment complex in the core of the city [24]. We aimed to obtain in total, approximately 4500 respondents (*i.e.*, 1125 respondents for each survey strata). 

### 2.2. Survey and Noise Annoyance Data

The purpose of the survey was to evaluate perceived annoyance and sleep disturbance from various noise sources in the population living on the island of Montreal. The questionnaire was adapted from the European LARES-survey (Large Analysis and Review of European housing and health Status) [25]. Questions on socio-demographic factors (age, gender, household income, education, number of people per household, time lived at the place of residence) and self-estimated noise-sensitivity were also included. Postal codes that allow to geographically locating the respondents were verified during the interview. The survey was carried out as telephone interviews between the 10 April and the 20 June 2014. 

This article addresses only the results regarding annoyance due to transportation noise sources and the total (outdoor) environmental noise. Annoyance was assessed as noise-induced disturbance [26] in a five-point category scale for eight outdoor noise sources: (1) traffic; (2) trains; (3) airplanes; (4) parking lots; (5) neighborhood (bars, discos, demonstrations); (6) animals or birds (outdoors); (7) shopping centers, industrial or construction zones; and (8) schools and parks. The question was phrased as follows: “In the past 12 months, have you been not at all, a little, somewhat, quite a bit or a lot disturbed at home by the following sources of noise?” Note that we did not include the words “bothered” or “annoyed” in addition to “disturbed” in our question. In the presentation of the results, the “annoyed” category consists of those that were somewhat, quite a bit or a lot disturbed by a specific noise source, and the “highly annoyed” category included those who reported being quite a bit or a lot disturbed. Non-applicable answers were considered as not annoyed. High annoyance to total environmental noise was defined as being quite a bit or a lot disturbed by at least one of the outdoor noise sources (1 to 8). 

### 2.3. Noise Levels and Distance to Noise Sources

A-weighted outdoor noise levels (LA_eq24h_) and day-evening-night equivalent noise levels (L_den_) for 2014 were estimated at the geographic coordinate of the six digit postal code of each subject. L_den_ includes a 5 dB(A) penalty for the evening (19:00–22:59) and a 10 dB(A) penalty for the night-time hours (23:00–6:59). Total noise estimates were derived from land use regression (LUR) noise models developed by Ragettli *et al.* [18]. In brief, the LUR models for LA_eq24h_ and L_den_ were built based on 204 noise samples collected during two periods: a two-week sampling period in the summer 2010 [16], and a five-week sampling campaign in the spring 2014. Measurements were performed at all sites for at least one week. Noise levels were measured continuously in 2-min intervals (recording the 2-min averages) with the Type-II Sound Level Meter Data Logger Noise Sentry (Convergence Instruments, Sherbrooke, QC, Canada). LUR models were developed by establishing a statistical relationship between the noise measurements and surrounding determinants of the built and natural environment. The models explained 68% and 69% of the variability in environmental noise levels for LA_eq24h_ and L_den_, respectively. Among others, the models included predictor variables related to all traffic noise sources: road (length of major roads within 50 m, annual average traffic counts at the nearest road, number of intersections within 200 m, distance to highways), rail (presence of a railway within 150 m, presence of a railroad shunting yard within 100 m) and air traffic (1 km or less from the NEF25 contour). Main predictors of measured noise levels were road traffic and vegetation variables. A map of the estimated noise levels for Montreal can be found in [18]. In addition, for each subject, the distance of the residential six digit postal code to the nearest transportation noise source such as major roads, railways, and the NEF25 contour was computed using the open software PostGreSQL 9.1 (PostgreSQL Global Development Group, Berkeley, CA, USA). The distances to the closest major road and railway (which also includes the main railway lines of railroad shunting yards) were divided in 50 m categories based on the distributions [7]. The distance to the NEF25 contour was categorized by 1000 m (1–1000, 1001–2000, >2000), including a category *within the NEF25 contour* [22]. 

### 2.4. Survey Weights and Statistical Analysis

In order for noise annoyance estimates produced from the survey to be representative of the total population of Montreal (≥18 years), survey weights were computed for each record considering the survey strata (road, rail, NEF25 and non-exposed), and age, gender and education. First, we computed the sampling weights for each stratum by multiplying the reciprocals of the proportions of respondents by strata and the corresponding proportion of the total population aged 18 and more (obtained from the 2011 Census [19]). These sampling weights were then “raked” so that weighted totals would match census totals for sex, and nine classes of age and education (*i.e.*, three classes of education × three classes of age) [27]. 

The association between the percentage of “highly annoyed” (%HA) and “annoyed” persons (%A) (with a dichotomous response variable) due to single environmental noise sources (*i.e.*, traffic, trains, airplanes) and to total environmental noise sources, and continuous noise levels (*i.e.*, LA_eq24h_ and L_den_) were analyzed using log-binomial regression models. We used log-binomial regression models to obtain covariate-adjusted Prevalence Proportion Ratios (PPR) of noise annoyance in relation to noise levels as suggested by Barros *et al.*, 2003 [28]. Marginal proportions for annoyed and highly annoyed people in 5 dB(A) noise categories were derived from these regression models using the STATA command “margins”. Similarly, the annoyance due to traffic, trains and airplanes was studied in relation to distance (in categories) to the respective transportation source. All models were adjusted for age, sex and education (in categories described in Table 1).

Statistical analyses were conducted in STATA SE version 13.0 (StataCorp, College Station, TX, USA). 

## 3. Results 

In total, 15,697 randomly selected telephone numbers were dialed (of which 21.5% were out of service or not valid) to achieve 4500 respondents. Using the American Association for Public Opinion Research (AAPOR) standards [29], this corresponds to a response rate of 46.8%. After excluding ineligible observations (missing data for age and/or education for generating survey weights), 4336 observations were available for computing the survey weights and carrying out the analyses. The distribution of the respondents among the four survey strata road, rail, air and the non-exposed was 25.0%, 25.1%, 24.6% and 25.3%, respectively. The socio-demographic characteristics of the study sample with complete data, as well as the noise exposure categories in relation to distance to transportation noise source and estimated LA_eq24h_ noise levels are presented in Table 1 (corresponding population-weighted results are presented in the supplemental material, Table S1). The crude proportions of individuals of the study sample that have been living <5 years, 6–10 years and >10 years at their place of residence was 32.3%, 21.0% and 46.7%, respectively. 

Estimated environmental noise levels at postal codes ranged between 50.1–76.1 for LA_eq24h_ and between 55.0–78.7 dB(A) for L_den_. The majority of the study participants (48%) were exposed to noise levels (L_den_) between 61 and 65 dB(A) (see Table 1 for LA_eq24h_). 

**Figure 2:**
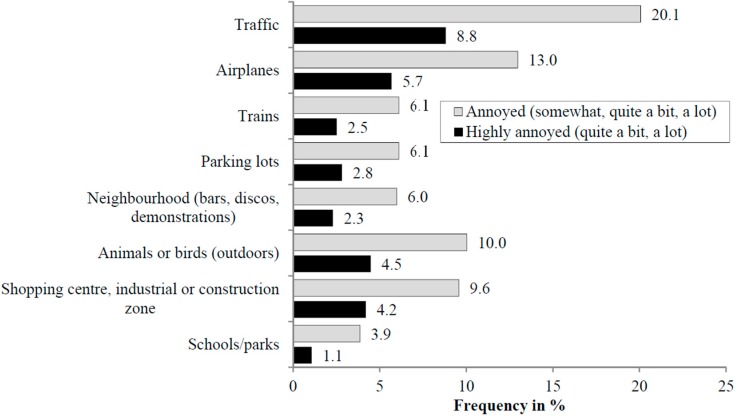
The proportion annoyed (somewhat, quite a bit, a lot) and highly annoyed (quite a bit, a lot) due to eight outdoor noise sources in the weighted study sample.

The weighted proportion of the population that was annoyed and highly annoyed by at least one environmental noise source (*i.e.*, with outdoor origin) was 42.3% and 23.1%, respectively. Figure 2 shows the population-weighted percentage of highly annoyed (%HA) and percentage of annoyed (%A) to the various noise sources. In Montreal, 20.1% of the population was annoyed by road traffic noise, followed by airplane noise with 13.0%. Noise from trains ranked seventh with 6.1% of the population. Similarly, the %HA was highest for road traffic (8.8%), followed by aircraft noise (5.7%). In the weighted sample, 18.1% of the population considered themselves to be quite a bit or a lot sensitive to noise. We did not detect a statistically significant difference in noise sensitivity between survey strata.

**Table 1:** Characteristics of the survey respondents (*n* = 4336). Results are not population-weighted.

Characteristics	%
Age categories	
18–29	6.9
30–39	13.5
40–49	19.1
50–59	23.3
60–69	19.9
70–80	11.6
>80	5.7
Sex	
Women	48.2
Men	51.8
Educational level	
No diploma or elementary school	8.0
High school	17.5
College	26.2
University degree	48.3
Distance of residential postal code to noise source (in m)	median (range)
Major road	148 (1–2043)
Railway	722 (5–4531)
NEF25 contour of Montreal International Airport	5887 (0–25,743)
Exposure by transportation noise source	
Road (within 100 m of a major road)	39.1
Airplanes (within 1000 m of NEF zone)	24.4
Rail (within 150 m of a railway)	19.1
Not-exposed	29.7
Estimated LA_eq24_ noise level *	
low (<55 dBA)	7.9
medium (56–60 dBA)	45.6
high (61–65 dBA)	34.8
very high (>65 dBA)	11.7

Note: * A Land Use Regression model was used to estimate the noise levels for the year 2014.

**Figure 3:**
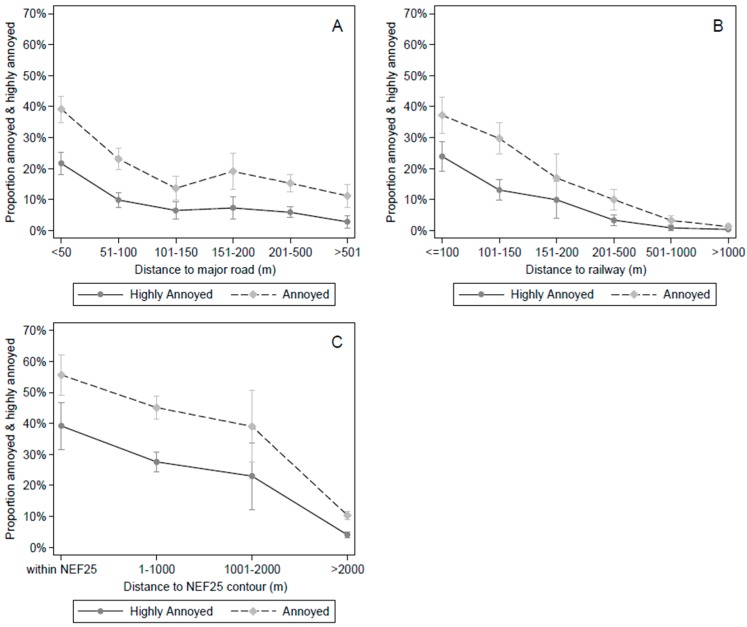
Estimated marginal proportions of annoyed and highly annoyed persons (with 95% CI) from road traffic (**A**); train (**B**) and airplane (**C**) noise by distance to the transportation sources (in categories), adjusted for age, education and sex.

We found a strong relationship between the distance to the noise source and the prevalence of annoyance from all transportation noise sources. Figure 3 shows the adjusted proportions with 95% confidence intervals of highly annoyed and annoyed persons predicted at various distance categories from the noise sources (log-binomial regression models are presented in the Supplement Tables S2–S7). For example, the adjusted %HA due to road traffic noise was 22% within 50 m, 10% within 51–100 m, and below 10% at categories of 100 and more meters away from major roads. Thus, a sharp decrease of %HA and %A due to road traffic noise was observed for the first 150 m. The %HA and %A due to noise from trains rapidly decreased when moving away from the railway tracks. We observed that a considerable proportion of the population was disturbed by aircraft noise, even outside of the NEF25 zone. In the residential areas between 1 and 2 km away from the NEF25 zone, the adjusted predictions of annoyance and high annoyance was still 39% and 23%, respectively. 

**Figure 4:**
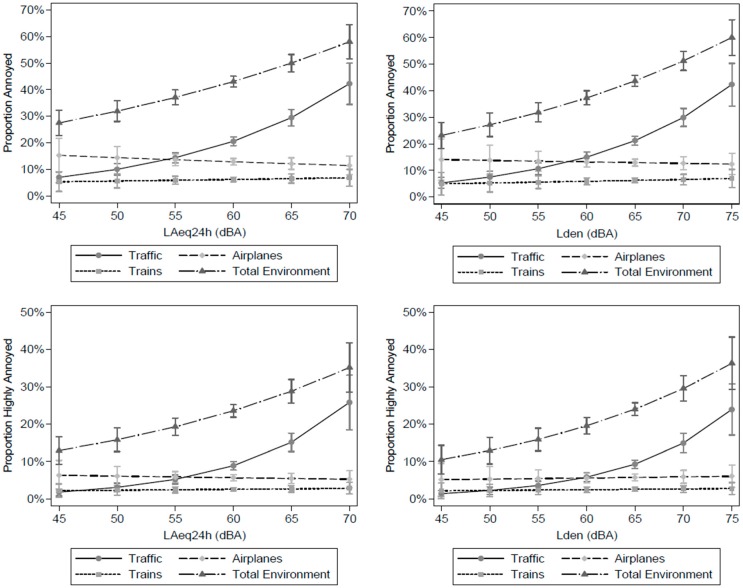
Estimated marginal proportions of annoyed (a lot, quite a bit, somewhat) and highly annoyed (a lot, quite a bit) persons in the total study population (with 95% CI) from traffic, airplanes, trains and total environmental noise (includes noise from transport, neighborhood, industrial and commercial sources, schools, parks, animals and birds) as a function of LA_eq24h_ (**left**) and L_den_ (**right**), adjusted for age, education and sex.

Figure 4 illustrates the exposure-response relationships for %HA and %A from road traffic, trains, airplanes and for annoyance from total environmental noise in relation to modeled LA_eq24h_ (left) and L_den_ (right) in the total study population. The adjusted marginal proportions of annoyance were predicted for 5 dB(A) noise categories. For %HA, we found a Prevalence Proportion Ratios of 1.10 (95% CI: 1.07–1.13) and 1.04 (1.02–1.06) per 1 dB(A) increase in L_den_ levels, for annoyance due to traffic noise and to total environmental noise, respectively (the log-binomial regression models for %A and %HA related to L_den_ and LA_eq24_ are provided in the supplemental material, Tables S8–S23). The adjusted proportion of the population highly annoyed by road traffic noise was 4% at an L_den_ of 55 dB(A) and reached 24% at an L_den_ of 75 dB(A). While we observed increasing proportions of %HA and %A from road traffic noise and the total environmental noise with both increasing LA_eq24h_ and L_den_ levels, no relationship between estimated environmental noise levels and annoyance from trains and airplane noise was found. Comparing the adjusted marginal proportion of highly annoyed people from total environmental noise in the noise sensitive (a lot, quite a bit) and non-sensitive (not at all, a bit, somewhat) subgroups showed that noise sensitivity increases annoyance independently by noise exposure level (Table S24). 

## 4. Discussion

We assessed the prevalence of noise annoyance in Montreal, Canada, in relation to distance to transportation noise sources and to estimated environmental noise levels. The most common outdoor noise sources of annoyance in the population were road traffic noise, followed by airplane noise. We observed a clear relationship between the distance to transportation noise sources and the prevalence of noise annoyance caused by road traffic, trains and airplanes. As the distance to major roads, railways and NEF25 zone of the Montreal International airport increased, %HA and %A due to the corresponding traffic noise gradually decreased. However, we noted a relationship with total noise levels (*i.e.*, LA_eq24h_ and L_den_) only with annoyance from road traffic and from total environmental noise sources. 

Our results are consistent with other studies that showed that the prevalence of annoyance and high annoyance is higher close to major roads, railways and airports than further away [7,17,30,31]. For instance, a national Canadian study on road traffic noise annoyance in 2005 found that people living within a self-reported proximity of 30 m to a heavily traveled road were more likely to indicate that they were highly annoyed by traffic noise than those who lived 30–500 and >500 m away. Compared to people living >500 m away from busy roads, respondents living next to a busy road were 6.0 times more likely to be bothered by road traffic noise [17]. The %HA and %A in the close proximity to railways and major roads was lower in Montreal than in previous studies conducted elsewhere in the world. For instance, in the Swedish municipality of Lerum, the %A within 101–150 m of a railway was 50% while it was 30% in Montreal. Possible explanations for the higher prevalence in the latter study compared to Montreal are the higher train frequencies in Lerum and the fact that all study participants lived relatively close to the railway (0–800 m in Lerrum *versus* 5–4531 m in Montreal) [7]. In addition, differences in %HA and %A may also arise due to differences in train type frequencies (passenger trains and freight trains), railway infrastructure and train speed [32,33]. Similarly to previous studies, we found a significant exposure-response relationship between noise levels and annoyance induced by road traffic noise. However, comparing our exposure-response curves to similar produced functions of %HA and %A as for example in [4,5,6,34], we generally observed lower percentages of annoyance for the same level of L_den_ in Montreal. For instance, applying the established equation for %HA due to road traffic noise by Miedema and Oudshoorn [5] that is based on several studies, the %HA at a L_den_ of 55 dB(A) and 75 dB(A) were 6.4% and 36.7%, respectively. In Montreal, the corresponding percentages were 3.6% and 23.9%. In fact, the noise level in L_den_ at which more than 10% of the population was highly annoyed by road traffic noise was 5 dB(A) lower (and outside the 95% CI) in Montreal than presented in the synthesis curves by Miedema and Oudshoorn [5]. Unlike other studies, we did not find a clear relationship between noise levels and annoyance from trains and airplanes (although associations were found with distance from these sources). 

There are a number of considerations related to the methods and the characteristics of the built environment that may explain the observed differences in the exposure-response relationship between annoyance and noise levels in our study compared to previous studies. First, we used LUR models developed based on the association between noise measurements of the total outdoor sound environment and land use characteristics of the built and natural environment. Previous studies mostly assessed the dose-response relationship for annoyance with numerical models that predict noise for a specific source by means of physical rules of noise propagation and attenuation [4,5,7,35,36]. LUR models may be unable to assess small-scale variations of noise levels due to physical characteristics of noise (e.g., sound reflection from the built environment). In fact, LUR modeling represents a rather new method to model noise in epidemiological studies. The LUR models used in this study explained close to 70% of the spatial variability of environmental noise levels in Montreal [18]. They could potentially be improved by including more detailed information on the road and railway networks as well as airplane and train frequencies. Nonetheless, LUR models have been suggested as a promising tool for exposure assessment in epidemiological studies, especially for areas where high quality numerical models are not available. A recent comparison of three noise LUR models—that were built with 20-min short-term noise measurements to estimates within-city variability of road traffic noise in three European cities—to standard noise models showed no systematic differences in the spatial patterns between LUR estimates and noise estimates from standard noise models. However, LUR estimates tended to be higher than those of standard models, especially at low noise levels [37]. Additionally, propagation noise models may have limitations in accurately representing the exposure situation encountered by populations. Comparisons of such noise propagation estimates with continuous noise measurements have shown systematic underestimation of modeled noise levels, partly as models neglect local noise sources such as for example parking cars [11,38]. Further comparisons between noise propagation models and LUR models for various transportation noise sources, and potential differences when using them in health assessments are warranted. 

Second, it needs to be considered that the noise estimates used in our study are from noise LUR models which themselves present limitations [16,37]. For example, noise levels were measured using Type-II devices which do not accurately measure noise levels below 40 dB(A) and although corrected, sound reflection from buildings cannot be ruled out. Most importantly, the models were built based on 2-min average noise levels. It has been shown that there can be large differences in maximum noise levels between road traffic and railway noise, even if the total sound levels are the same [7]. Thus, in contrast to the rather constant noise from road traffic, the sampling interval may be too large to capture the intermittent noise produced by trains or airplanes passing by. This may explain why we did not find a relationship between noise levels (LA_eq24h_ and L_den_) and annoyance from trains and airplanes. Furthermore, it may be that instead of total environmental noise, only noise from railway and aircrafts, which we did not model, is related to annoyance from these sources. Alternatively, road traffic noise that contributes to environmental noise in proximity to railways and airports may contribute more to LA_eq24h_ than noise from these sources and may also partly explain why we did not find a clear relationship between total environmental noise levels and annoyance from railway and aircraft noise. To our knowledge, the noise LUR models by Ragettli *et al.* [18] are the first aiming to include also noise from rail and air traffic noise. To better understand the insignificant relationship between noise levels and annoyance from trains and airplanes found in this study, further investigations are needed that formally compare noise exposure estimates close to railways and airports from LUR models to those of propagation models using noise measurements of high temporal resolution. Additionally, it is recommended that further studies on transportation noise induced annoyance take into account differences in acoustic properties between the noise sources. 

Finally, it should be emphasized that most of the studies on transportation noise induced annoyance were conducted in Europe, where cities are frequently configured differently than in North America. For example, the distance of the buildings relative to the street and the street width is generally larger in Montreal than in many European cities, in particular in residential areas. This may also contribute to the lower prevalence of annoyance in close proximity to major roads observed in Montreal compared to other cities [7,30]. In addition, due to the cold winter temperatures in Montreal, houses are isolated well and windows—that are often double or triple glazed—are normally closed during winter months, which likely also contributes to lower perceived disturbance from traffic noise during the year [2,9]. However, a cold winter climate may not solely lead to lower noise annoyance prevalence as for example a Norwegian study [34] found stronger exposure-response relationships between road-traffic noise and indoor noise annoyance than reported in our study and in Miedema and Oudshoorn [5]. Despite the methodological differences, this would imply that the noise annoyance prevalence and the exposure-response relationship are variable between cities and continents and are dependent on local characteristics such as the built environment and the characteristics of the traffic noise sources. This is contrary to the hypothesis from Miedema and Oudshoorn [5] who hypothesized that there are no important differences between countries in the reaction of the population to similar noise exposures and future work is needed to better characterize noise exposure differences between cities, countries and continents. 

To our knowledge, this is the first comprehensive study assessing the prevalence of annoyance to various transportation noise sources in a North American city, where policies regulating noise are less prevalent and noise-related health effects are less studied than in European countries [11,12]. Unlike previous studies establishing exposure-response curves for noise annoyance, we related annoyance to noise levels that are based on actual noise measurements. 

A limitation of our study is the lack of information on additional exposure modifiers and exposure misclassification. Noise exposure was assessed by modeled noise levels and distances to transportation noise sources based on residential postal codes only. Thus, possible misclassification of exposure cannot be ruled out as information on the position of the main living quarters relative to the road or railway and on façade insulation could not be considered [2,9,39]. It is important to note though that our focus was on the community response, not on individuals. As we assessed prevalence of annoyance, we did not have the information on whether people moved to a quieter area of the city due to noise annoyance. The fact that the prevalence of people reporting to be a lot or quite a bit sensitive to noise is similar in all survey strata suggests that people easily annoyed by noise did not necessarily live in quieter areas [40].

## 5. Conclusions 

Our study provides the first comprehensive information on the relation between transportation noise sources, total environmental noise levels and prevalence of noise annoyance in Montreal. Our study clearly shows that Montreal residents living near busy roads, main railway lines, as well as within and close to the NEF25 zone of the Montreal International airport are annoyed by transportation noise. 

There is increasing evidence that exposure to environmental noise is related—in addition to annoyance—to various negative health outcomes such sleep problems, impaired cognitive performance, hypertension and cardiovascular disease [1]. Since national approaches to assess noise exposure currently do not exist in Canada, initiatives aiming to reduce and monitor noise exposure in Canadian cities are warranted at various levels of government. Adoption of such directives would require the specification, for example, of allowed construction zones around traffic sources and acceptable methods for noise monitoring and modelling. 

## Supplementary Files

Supplementary File 1

## References

[1] Basner M., Babisch W., Davis A., Brink M., Clark C., Janssen S., Stansfeld S. (2014). Auditory and non-auditory effects of noise on health. Lancet.

[2] Öhrström E., Skånberg A., Svensson H., Gidlöf-Gunnarsson A. (2006). Effects of road traffic noise and the benefit of access to quietness. J. Sound Vib..

[3] Dratva J., Zemp E., Dietrich D.F., Bridevaux P.-O., Rochat T., Schindler C., Gerbase M.W. (2010). Impact of road traffic noise annoyance on health-related quality of life: Results from a population-based study. Qual. Life Res..

[4] Heritier H., Vienneau D., Frei P., Eze I.C., Brink M., Probst-Hensch N., Roeoesli M. (2014). The association between road traffic noise exposure, annoyance and health-related quality of life (HRQOL). Int. J. Environ. Res. Public Health.

[5] Miedema H.M.E., Oudshoorn C.G.M. (2001). Annoyance from transportation noise: Relationships with exposure metrics DNL and DENL and their confidence intervals. Environ. Health Perspect..

[6] European Commission (2002). Position paper on Dose Response Relationships between Transportation Noise and Annoyance.

[7] Öhrstrom E., Barregård L., Andersson E., Skånberg A., Svensson H., Ängerheim P. (2007). Annoyance due to single and combined sound exposure from railway and road traffic. J. Acoust. Soc. Am..

[8] WHO (2011). Burden of disease from environmental noise. Quantification of Healthy Life Years Lost in Europe.

[9] Babisch W., Swart W., Houthuijs D., Selander J., Bluhm G., Pershagen G., Dimakopoulou K., Haralabidis A.S., Katsouyanni K., Davou E. (2012). Exposure modifiers of the relationships of transportation noise with high blood pressure and noise annoyance. J. Acoust. Soc. Am..

[10] European Commission (2002). Directive 2002/49/EC of the European Parliment and of the Council of 25 June 2001 relating to the assessment and management of environmental noise. Off. J. Eur. Commun..

[11] Lee E.Y., Jerrett M., Ross Z., Coogan P.F., Seto E.Y.W. (2014). Assessment of traffic-related noise in three cities in the United States. Environ. Res..

[12] Gan W.Q., McLean K., Brauer M., Chiarello S.A., Davies H.W. (2012). Modeling population exposure to community noise and air pollution in a large metropolitan area. Environ. Res..

[13] Steele C. (2001). A critical review of some traffic noise prediction models. Appl. Acoust..

[14] King G., Roland-Mieszkowski M., Jason T., Rainham D.G. (2012). Noise levels associated with urban land use. J. Urban. Health Bull. N. Y. Acad. Med..

[15] Zuo F., Li Y., Johnson S., Johnson J., Varughese S., Copes R., Liu F., Wu H.J., Hou R., Chen H. (2014). Temporal and spatial variability of traffic-related noise in the city of Toronto, Canada. Sci. Total Environ..

[16] Goudreau S., Plante C., Fournier M., Brand A., Roche Y., Smargiassi A. (2014). Estimation of spatial variations in urban noise levels with a land use regression model. Environ. Pollut..

[17] Michaud D.S., Keith S.E., McMurchy D. (2008). Annoyance and disturbance of daily activities from road traffic noise in Canada. J. Acoust. Soc. Am..

[18] Ragettli M.S., Goudreau S., Plante C., Fournier M., Hatzopoulou M., Perron S., Smargiassi A. (2015). Statistical modeling of the spatial variability of environmental noise levels in Montreal, Canada, using noise measurements and land use characteristics. J. Expos. Sci. Environ. Epidemiol..

[19] Statistics Canada Population and Dwelling Counts, for Canada and Economic Regions, 2011 and 2006 Censuses. https://www12.statcan.gc.ca/census-recensement/2011/dp-pd/hlt-fst/pd-pl/Table-Tableau.cfm?LANG=Eng&T=1401&SR=1&S=9&O=D&RPP=25.

[20] Transport Canada Noise Exposure Forecast and Related Programs. https://www.tc.gc.ca/eng/civilaviation/standards/aerodromeairnav-standards-noise-nef-924.htm.

[21] BOING Courbe D’ambiance Sonore. http://www.boeing.com/commercial/noise/montreal2011.pdf.

[22] Dale L.M., Debia M., Mudaheranwa O.C., Plante C., Smargiassi A. (2013). An exploration of transportation source contribution to noise levels near an airport. Environ. Pollut..

[23] Price K., Perron S. (2013). Avis de Santé Publique Concernant Les Impacts Sanitaires du Bruit Engendré Par Les Activités Ferroviaires de la Compagnie CN à Pointe-Saint-Charles.

[24] Statistics Canada Postal Code Conversion File (PCCF), Reference Guide. http://geodepot.statcan.ca/2006/Reference/Freepub/92-153-GWE/2007002/using.htm.

[25] Niemann H., Bonnefoy X., Braubach M., Hecht K., Maschke C., Rodrigues C., Robbel N. (2006). Noise-induced annoyance and morbidity results from the pan-European LARES study. Noise Health.

[26] Guski R., Felscher-Suhr U., Schuemer R. (1999). The concept of noise annoyance: How international experts see it. J. Sound Vib..

[27] Frankel M. (2010). Sampling theory. Handbook of Survey Research.

[28] Barros A.J., Hirakata V.N. (2003). Alternatives for logistic regression in cross-sectional studies: An empirical comparison of models that directly estimate the prevalence ratio. BMC Med. Res. Methodol..

[29] American Association for Public Opinion Research (AAPOR) Response Rates—An Overview. http://www.aapor.org/AAPORKentico/Education-Resources/For-Researchers/Poll-Survey-FAQ/Response-Rates-An-Overview.aspx.

[30] Di G., Liu X., Lin Q., Zheng Y., He L. (2012). The relationship between urban combined traffic noise and annoyance: An investigation in Dalian, north of China. Sci. Total Environ..

[31] Morihara T., Sato T., Yano T. (2004). Comparison of dose-response relationships between railway and road traffic noises: The moderating effect of distance. J. Sound Vib..

[32] Pennig S., Quehl J., Mueller U., Rolny V., Maass H., Basner M., Elmenhorst E.-M. (2012). Annoyance and self-reported sleep disturbance due to night-time railway noise examined in the field. J. Acoust. Soc. Am..

[33] Gidlof-Gunnarsson A., Ogren M., Jerson T., Ohrstrom E. (2012). Railway noise annoyance and the importance of number of trains, ground vibration, and building situational factors. Noise Health.

[34] Klæboe R., Amundsen A., Fyhri A., Solberg S. (2004). Road traffic noise—The relationship between noise exposure and noise annoyance in Norway. Appl. Acoust..

[35] Janssen S.A., Vos H., van Kempen E.E.M.M., Breugelmans O.R.P., Miedema H.M.E. (2011). Trends in aircraft noise annoyance: The role of study and sample characteristics. J. Acoust. Soc. Am..

[36] Klaeboe R. (2011). Noise and health: Annoyance and interference. Encyclopedia of Environmental Health.

[37] Aguilera I., Foraster M., Basagaña X., Corradi E., Deltell A., Morelli X., Phuleria H.C., Ragettli M.S., Rivera M., Thomasson A. (2015). Application of land use regression modelling to assess the spatial distribution of road traffic noise in three European cities. J. Expo. Sci. Environ. Epidemiol..

[38] Mioduszewski P., Ejsmont J.A., Grabowski J., Karpiński D. (2011). Noise map validation by continuous noise monitoring. Appl. Acoust..

[39] Eriksson C., Nilsson M.E., Stenkvist D., Bellander T., Pershagen G. (2013). Residential traffic noise exposure assessment: Application and evaluation of European Environmental Noise Directive maps. J. Expo. Sci. Environ. Epidemiol..

[40] Okokon E.O., Turunen A.W., Ung-Lanki S., Vartiainen A.-K., Tiittanen P., Lanki T. (2015). Road-traffic noise: Annoyance, risk perception, and noise sensitivity in the Finnish adult population. Int. J. Environ. Res. Public Health.

