# Supplementary material for: Annoyance from Road Traffic, Trains, Airplanes and from Total Environmental Noise Levels
Source: Int J Environ Res Public Health. 2015 Dec 29;13(1):90. doi: 10.3390/ijerph13010090 (PMC4730481; doi:10.3390/ijerph13010090)
Supplement: Supplementary File 1 [file ijerph-13-00090-s001.pdf]

Supplementary Materials: Annoyance from Road Traffic, Trains, Airplanes and from Total Environmental Noise Levels

Martina S. Ragettli, Sophie Goudreau, Céline Plante, Stéphane Perron, Michel Fournier and Audrey Smargiassi

Table S1. Summary statistics of population-weighted study sample.

Characteristics	%
Age categories	
18–29	11.7
30–39	13.1
40–49	18.8
50–59	25.1
60–69	18.8
70–80	8.1
>80	4.3
Sex	
Women	47.0
Men	53.0
Educational level	
No diploma or elementary school	11.0
High school	25.7
Cegep	26
University degree	37.2
Distance of postal code to noise source (in m)	mean (SD)
Major road	246.2 (4.8)
Railway	1122.0 (18.5)
NEF25 curve	8250.9 (100.6)
Exposure by transportation noise source	
Road (within 100 m of a major road)	33.4
Airplanes (within 1000 m of NEF zone)	4.8
Rail (within 150 m of a railway)	7.3
None	58.2
estimated LAeq24 noise level *	
low (<55 dBA)	12.7
medium (56–60 dBA)	47.3
high (61–65 dBA)	31.1
very high (>65 dBA)	8.9

Note: * A Land Use Regression model was used to estimate the noise levels for the year 2014.

Table S2. Log-binomial regression models for the percentage of highly annoyed people (a lot, quite a bit) due to road traffic in relation to the distance to major roads.

Variable	PPR [1]	SE	*p*-Value	(95% CI)
Distance (m)				
>50	1			
51–100	0.454	0.069	0.000	(0.337, 0.613)
101–150	0.296	0.071	0.000	(0.185, 0.474)
151–200	0.334	0.092	0.000	(0.195, 0.573)
201–500	0.268	0.047	0.000	(0.190, 0.378)
>501	0.127	0.047	0.000	(0.061, 0.264)
Age				
18–29	1			
30–39	0.957	0.288	0.883	(0.530, 1.727)
40–49	1.265	0.366	0.416	(0.718, 2.231)
50–59	1.531	0.440	0.138	(0.872, 2.690)
60–69	1.196	0.362	0.554	(0.661, 2.163)
70–79	1.009	0.341	0.980	(0.519, 1.959)
≥80	0.682	0.295	0.376	(0.293, 1.591)
Education				
No diploma	1			
Highschool	0.658	0.176	0.117	(0.390, 1.111)
Cegep	0.951	0.233	0.839	(0.589, 1.538)
University degree	0.872	0.202	0.555	(0.553, 1.374)
Sex				
Men	1			
Women	1.115	0.140	0.386	(0.872, 1.425)
Constant	0.199	0.073	0.000	(0.097, 0.410)

Note: [1] PPR: Prevalence Proportion Ratio; SE: Standard Error.

Table S3. Log-binomial regression models for the percentage of annoyed people (a lot, quite a bit, somewhat) due to road traffic in relation to the distance to major roads.

Variable	PPR	SE	*p*-Value	(95% CI)
Distance (m)				
>50	1			
51–100	0.589	0.056	0.000	(0.489, 0.709)
101–150	0.347	0.054	0.000	(0.256, 0.471)
151–200	0.487	0.080	0.000	(0.353, 0.671)
201–500	0.389	0.041	0.000	(0.315, 0.479)
>501	0.284	0.052	0.000	(0.199, 0.406)
Age				
18–29	1			
30–39	1.164	0.214	0.411	(0.811, 1.669)
40–49	1.245	0.222	0.219	(0.878, 1.765)
50–59	1.379	0.242	0.067	(0.978, 1.945)
60–69	1.131	0.208	0.506	(0.788, 1.623)

Table S2. *Cont.*

Variable	PPR	SE	*p*-Value	(95% CI)
Age				
70–79	0.870	0.184	0.509	(0.575, 1.316)
≥80	0.928	0.243	0.776	(0.556, 1.551)
Education				
No diploma	1			
Highschool	1.014	0.188	0.938	(0.706, 1.457)
Cegep	1.219	0.215	0.262	(0.863, 1.721)
University degree	1.293	0.218	0.128	(0.929, 1.801)
Sex				
Men	1			
Women	0.976	0.075	0.755	(0.839, 1.135)
Constant	0.288	0.068	0.000	(0.181, 0.458)

Table S4. Log-binomial regression models for the percentage of highly annoyed people (a lot, quite a bit) due to trains in relation to the distance to railways.

Variable	PPR	SE	*p*-Value	(95% CI)
Distance (m)				
>100	1			
101–150	0.546	0.089	0.000	(0.396, 0.753)
151–200	0.413	0.135	0.007	(0.217, 0.784)
201–500	0.138	0.042	0.000	(0.076, 0.251)
501–1000	0.032	0.017	0.000	(0.011, 0.092)
>1000	0.013	0.005	0.000	(0.006, 0.029)
Age				
18–29	1			
30–39	2.368	0.941	0.030	(1.086, 5.16)
40–49	1.793	0.705	0.138	(0.829, 3.874)
50–59	1.603	0.660	0.252	(0.714, 3.595)
60–69	1.690	0.643	0.168	(0.801, 3.565)
70–79	1.878	0.827	0.152	(0.793, 4.451)
≥80	2.487	1.151	0.049	(1.003, 6.162)
Education				
No diploma	1			
Highschool	1.148	0.456	0.728	(0.527, 2.500)
Cegep	1.744	0.657	0.140	(0.834, 3.648)
University degree	1.404	0.520	0.360	(0.679, 2.903)
Sex				
Men	1			
Women	0.823	0.147	0.274	(0.580, 1.167)
Constant	0.109	0.052	0.000	(0.043, 0.278)

Table S5. Log-binomial regression models for the percentage of annoyed people (a lot, quite a bit, somewhat) due to trains in relation to the distance to railways.

Variable	PPR	SE	*p*-Value	(95% CI)
Distance (m)				
>100	1			
101–150	0.799	0.096	0.062	(0.631, 1.011)
151–200	0.454	0.110	0.001	(0.283, 0.730)
201–500	0.267	0.056	0.000	(0.177, 0.403)
501–1000	0.086	0.022	0.000	(0.053, 0.141)
>1000	0.033	0.010	0.000	(0.017, 0.061)
Age				
18–29	1			
30–39	0.878	0.227	0.614	(0.528, 1.458)
40–49	0.894	0.231	0.663	(0.539, 1.482)
50–59	0.885	0.223	0.628	(0.540, 1.451)
60–69	0.616	0.164	0.069	(0.365, 1.038)
70–79	0.621	0.194	0.128	(0.337, 1.146)
≥80	0.769	0.280	0.470	(0.377, 1.568)
Education				
No diploma	1			
Highschool	1.147	0.345	0.649	(0.636, 2.069)
Cegep	1.235	0.369	0.480	(0.688, 2.218)
University degree	1.445	0.414	0.200	(0.823, 2.535)
Sex				
Men	1			
Women	0.986	0.128	0.916	(0.765, 1.272)
Constant	0.359	0.119	0.002	(0.187, 0.688)

Table S6. Log-binomial regression models for the percentage of highly annoyed people (a lot, quite a bit) due to airplanes in relation to the distance to the NEF25 contour of the Montreal International Airport.

Variable	PPR	SE	*p*-Value	(95% CI)
Distance (m)				
within NEF25	1			
1–1000	0.704	0.074	0.001	(0.573, 0.864)
1001–2000	0.586	0.147	0.033	(0.359, 0.958)
>2000	0.102	0.015	0.000	(0.076, 0.136)
Age				
18–29	1			
30–39	2.817	1.434	0.042	(1.038, 7.641)
40–49	3.382	1.610	0.010	(1.331, 8.599)
50–59	3.843	1.796	0.004	(1.537, 9.607)
60–69	2.402	1.149	0.067	(0.940, 6.137)
70–79	2.259	1.085	0.090	(0.881, 5.792)
≥80	1.262	0.694	0.672	(0.429, 3.710)

Table S6. *Cont.*

Variable	PPR	SE	*p*-Value	(95% CI)
Education				
No diploma	1			
Highschool	0.980	0.392	0.960	(0.448, 2.146)
Cegep	1.116	0.438	0.780	(0.517, 2.410)
University degree	1.024	0.401	0.952	(0.475, 2.205)
Sex				
Men	1			
Women	1.538	0.218	0.002	(1.165, 2.030)
Constant	0.106	0.059	0.000	(0.036, 0.314)

Table S7. Log-binomial regression models for the percentage of annoyed people (a lot, quite a bit, somewhat) due to airplanes in relation to the distance to the NEF25 contour of the Montreal International Airport.

Variable	PPR	SE	*p*-Value	(95% CI)
Distance (m)				
within NEF25	1			
1–1000	0.811	0.048	0.000	(0.722, 0.910)
1001–2000	0.701	0.110	0.024	(0.515, 0.954)
>2000	0.185	0.016	0.000	(0.156, 0.219)
Age				
18–29	1			
30–39	2.046	0.552	0.008	(1.207, 3.471)
40–49	2.471	0.621	0.000	(1.509, 4.045)
50–59	2.321	0.588	0.001	(1.413, 3.814)
60–69	1.603	0.422	0.073	(0.957, 2.686)
70–79	1.705	0.464	0.050	(1.000, 2.908)
≥80	1.002	0.334	0.996	(0.521, 1.926)
Education				
No diploma	1			
Highschool	1.246	0.343	0.423	(0.727, 2.136)
Cegep	1.658	0.442	0.058	(0.982, 2.797)
University degree	1.771	0.473	0.033	(1.049, 2.990)
Sex				
Men	1			
Women	1.160	0.097	0.076	(0.984, 1.367)
Constant	0	0.057	0.000	(0.092, 0.330)

Table S8. Log-binomial regression models for the percentage of highly annoyed people (a lot, quite a bit) due to road traffic noise as a function of modelled noise levels of the total sound environment in L_{den}.

Variable	PPR	SE	*p*-Value	(95% CI)
L_{den}	1.099	0.016	0.000	(1.069, 1.131)
Age				
18–29	1			
30–39	0.877	0.271	0.746	(0.479, 1.608)
40–49	1.167	0.348	0.551	(0.650, 2.094)
50–59	1.437	0.424	0.214	(0.806, 2.564)
60–69	1.071	0.333	0.833	(0.582, 1.970)
70–79	0.938	0.329	0.424	(0.471, 1.866)
≥80	0.643	0.284	0.413	(0.270, 1.529)
Education				
No diploma	1			
Highschool	0.675	0.184	0.200	(0.395, 1.151)
Cegep	0.996	0.251	0.636	(0.608, 1.631)
University degree	0.951	0.226	0.662	(0.597, 1.517)
Sex				
Men	1			
Women	1.069	0.137	0.130	(0.831, 1.375)
Constant	0.000	0.000	0.0000	(0.000, 0.001)

Table S9. Log-binomial regression models for the percentage of annoyed people (a lot, quite a bit, somewhat) due to road traffic noise as a function of modelled noise levels of the total sound environment in L_{den}.

Variable	PPR	SE	*p*-Value	(95% CI)
L_{den}	1.072	0.010	0.000	(1.052, 1.091)
Age				
18–29	1			
30–39	1.103	0.212	0.609	(0.757, 1.608)
40–49	1.159	0.215	0.425	(0.806, 1.667)
50–59	1.323	0.241	0.124	(0.926, 1.89)
60–69	1.042	0.199	0.829	(0.716, 1.517)
70–79	0.839	0.184	0.423	(0.546, 1.289)
≥80	0.890	0.241	0.667	(0.523, 1.514)
Education				
No diploma	1			
Highschool	1.024	0.196	0.900	(0.704, 1.49)
Cegep	1.275	0.231	0.181	(0.893, 1.82)
University degree	1.391	0.242	0.058	(0.989, 1.957)
Sex				
Men	1			
Women	0.948	0.075	0.500	(0.811, 1.107)
Constant	0.002	0.001	0.000	(0.001, 0.006)

Table S10. Log-binomial regression models for the percentage of highly annoyed people (a lot, quite a bit) due to road traffic noise as a function of modelled noise levels of the total sound environment in LA_{eq24h}.

Variable	PPR	SE	*p*-Value	(95% CI)
LA_{eq24h}	1.113	0.017	0.000	(1.080, 1.147)
Age				
18–29	1			
30–39	0.886	0.269	0.690	(0.489, 1.606)
40–49	1.176	0.346	0.582	(0.660, 2.093)
50–59	1.482	0.433	0.178	(0.836, 2.628)
60–69	1.085	0.332	0.790	(0.596, 1.976)
70–79	0.936	0.327	0.850	(0.472, 1.855)
≥80	0.651	0.286	0.329	(0.275, 1.540)
Education				
No diploma	1			
Highschool	0.653	0.178	0.119	(0.382, 1.115)
Cegep	0.976	0.247	0.925	(0.594, 1.604)
University degree	0.956	0.228	0.849	(0.598, 1.526)
Sex				
Men	1			
Women	1.067	0.136	0.611	(0.831, 1.370)

Table S11. Log-binomial regression models for the percentage of annoyed people (a lot, quite a bit, somewhat) due to road traffic noise as a function of modelled noise levels of the total sound environment in LA_{eq24h}.

Variable	PPR	SE	*p*-Value	(95% CI)
LA_{eq24h}	1.075	0.010	0.000	(1.055, 1.095)
Age				
18–29	1			
30–39	1.107	0.210	0.593	(0.763, 1.606)
40–49	1.162	0.213	0.413	(0.811, 1.665)
50–59	1.340	0.242	0.105	(0.941, 1.908)
60–69	1.048	0.199	0.805	(0.722, 1.520)
70–79	0.839	0.183	0.422	(0.547, 1.288)
≥80	0.902	0.243	0.701	(0.532, 1.529)
Education				
No diploma	1			
Highschool	1.006	0.193	0.976	(0.691, 1.464)
Cegep	1.261	0.229	0.202	(0.883, 1.800)
University degree	1.390	0.242	0.059	(0.988, 1.956)
Sex				
Men	1			
Women	0.949	0.075	0.507	(0.813, 1.108)
Constant	0.002	0.001	0.000	(0.001, 0.007)

Table S12. Log-binomial regression models for the percentage of highly annoyed people (a lot, quite a bit) due to train noise as a function of modelled noise levels of the total sound environment in L_{den}.

Variable	PPR	SE	*p*-Value	(95% CI)
L_{den}	1.008	0.025	0.735	(0.960, 1.059)
Age				
18–29	1			
30–39	2.221	0.955	0.063	(0.956, 5.161)
40–49	1.384	0.585	0.442	(0.604, 3.169)
50–59	1.305	0.578	0.547	(0.548, 3.109)
60–69	1.230	0.496	0.608	(0.558, 2.711)
70–79	1.480	0.695	0.404	(0.589, 3.718)
≥80	1.678	0.863	0.315	(0.612, 4.600)
Education				
No diploma	1			
Highschool	1.265	0.548	0.588	(0.540, 2.959)
Cegep	1.422	0.580	0.388	(0.640, 3.163)
University degree	1.163	0.470	0.709	(0.526, 2.567)
Sex				
Men	1			
Women	0.756	0.152	0.164	(0.509, 1.121)
Constant	0.010	0.016	0.005	(0.000, 0.245)

Table S13. Log-binomial regression models for the percentage of annoyed people (a lot, quite a bit, somewhat) due to train noise as a function of modelled noise levels of the total sound environment in L_{den}.

Variable	PPR	SE	*p*-Value	(95% CI)
L_{den}	1.011	0.023	0.620	(0.967, 1.057)
Age				
18–29	1			
30–39	0.739	0.225	0.321	(0.406, 1.343)
40–49	0.688	0.206	0.211	(0.383, 1.236)
50–59	0.712	0.211	0.252	(0.398, 1.273)
60–69	0.434	0.128	0.005	(0.243, 0.774)
70–79	0.450	0.161	0.026	(0.223, 0.909)
≥80	0.549	0.231	0.154	(0.241, 1.252)
Education				
No diploma	1			
Highschool	1.138	0.383	0.700	(0.589, 2.201)
Cegep	1.001	0.336	0.998	(0.518, 1.933)
University degree	1.186	0.377	0.592	(0.636, 2.213)
Sex				
Men	1			
Women	0.967	0.147	0.822	(0.718, 1.301)
Constant	0.041	0.058	0.024	(0.003, 0.652)

Table S14. Log-binomial regression models for the percentage of highly annoyed people (a lot, quite a bit) due to train noise as a function of modelled noise levels of the total sound environment in LA_{eq24h}.

Variable	PPR	SE	*p*-Value	(95% CI)
LA_{eq24h}	1.010	0.028	0.707	(0.958, 1.066)
Age				
18–29	1			
30–39	2.218	0.959	0.065	(0.950, 5.177)
40–49	1.383	0.583	0.442	(0.605, 3.159)
50–59	1.306	0.577	0.545	(0.550, 3.104)
60–69	1.229	0.496	0.608	(0.558, 2.711)
70–79	1.479	0.696	0.406	(0.588, 3.718)
≥80	1.679	0.863	0.314	(0.613, 4.600)
Education				
No diploma	1			
Highschool	1.264	0.548	0.589	(0.540, 2.956)
Cegep	1.424	0.578	0.383	(0.643, 3.156)
University degree	1.167	0.475	0.705	(0.525, 2.593)
Sex				
Men	1			
Women	0.755	0.152	0.163	(0.509, 1.120)
Constant	0.009	0.015	0.005	(0.000, 0.233)

Table S15. Log-binomial regression models for the percentage of annoyed people (a lot, quite a bit, somewhat) due to train noise as a function of modelled noise levels of the total sound environment in LA_{eq24h}.

Variable	PPR	SE	*p*-Value	(95% CI)
LA_{eq24h}	1.010	0.023	0.670	(0.966, 1.055)
Age				
18–29	1			
30–39	0.739	0.225	0.322	(0.406, 1.344)
40–49	0.687	0.206	0.210	(0.382, 1.235)
50–59	0.711	0.211	0.250	(0.398, 1.272)
60–69	0.434	0.128	0.005	(0.243, 0.774)
70–79	0.450	0.161	0.026	(0.222, 0.909)
≥80	0.549	0.231	0.154	(0.241, 1.252)
Education				
No diploma	1			
Highschool	1.133	0.383	0.712	(0.584, 2.2)
Cegep	0.997	0.336	0.994	(0.515, 1.931)
University degree	1.182	0.378	0.600	(0.632, 2.213)
Sex				
Men	1			
Women	0.967	0.147	0.823	(0.718, 1.302)
Constant	0.048	0.063	0.022	(0.004, 0.641)

Table S16. Log-binomial regression models for the percentage of highly annoyed people (a lot, quite a bit) due to airplane noise as a function of modelled noise levels of the total sound environment in L_{den}.

Variable	PPR	SE	*p*-Value	(95% CI)
L_{den}	1.006	0.022	0.802	(0.963, 1.05)
Age				
18–29	1			
30–39	2.240	1.168	0.122	(0.806, 6.225)
40–49	2.993	1.445	0.023	(1.162, 7.711)
50–59	3.613	1.715	0.007	(1.425, 9.161)
60–69	2.103	1.032	0.130	(0.803, 5.506)
70–79	1.999	0.988	0.161	(0.759, 5.266)
≥80	1.305	0.740	0.638	(0.43, 3.965)
Education				
No diploma	1			
Highschool	1.123	0.457	0.775	(0.506, 2.494)
Cegep	1.299	0.523	0.516	(0.59, 2.862)
University degree	1.378	0.530	0.405	(0.648, 2.929)
Sex				
Men	1			
Women	1.475	0.227	0.012	(1.09, 1.995)
Constant	0.010	0.014	0.001	(0.001, 0.152)

Table S17. Log-binomial regression models for the percentage of annoyed people (a lot, quite a bit, somewhat) due to airplane noise as a function of modelled noise levels of the total sound environment in L_{den}.

Variable	PPR	SE	*p*-Value	(95% CI)
L_{den}	0.996	0.014	0.766	(0.968, 1.024)
Age				
18–29	1			
30–39	1.810	0.511	0.036	(1.041, 3.147)
40–49	2.390	0.627	0.001	(1.429, 3.998)
50–59	2.235	0.589	0.002	(1.332, 3.748)
60–69	1.465	0.401	0.163	(0.856, 2.507)
70–79	1.598	0.461	0.104	(0.908, 2.812)
≥80	1.040	0.364	0.910	(0.524, 2.067)
Education				
No diploma	1			
Highschool	1.299	0.364	0.351	(0.750, 2.251)
Cegep	1.796	0.488	0.031	(1.053, 3.061)
University degree	2.148	0.563	0.004	(1.284, 3.592)
Sex				
Men	1			
Women	1.094	0.108	0.367	(0.900, 1.328)
Constant	0.052	0.050	0.002	(0.008, 0.348)

Table S18. Log-binomial regression models for the percentage of highly annoyed people (a lot, quite a bit) due to airplane noise as a function of modelled noise levels of the total sound environment in LA_{eq24h}.

Variable	PPR	SE	*p*-Value	(95% CI)
LA_{eq24h}	0.993	0.021	0.719	(0.953, 1.034)
Age				
18–29	1			
30–39	2.253	1.175	0.119	(0.811, 6.264)
40–49	2.976	1.437	0.024	(1.155, 7.670)
50–59	3.585	1.701	0.007	(1.414, 9.088)
60–69	2.101	1.031	0.131	(0.803, 5.497)
70–79	1.987	0.983	0.165	(0.753, 5.241)
≥80	1.299	0.736	0.645	(0.427, 3.947)
Education				
No diploma	1			
Highschool	1.113	0.455	0.794	(0.499, 2.480)
Cegep	1.283	0.520	0.538	(0.580, 2.840)
University degree	1.347	0.523	0.443	(0.629, 2.885)
Sex				
Men	1			
Women	1.476	0.227	0.011	(1.092, 1.996)
Constant	0.023	0.029	0.002	(0.002, 0.265)

Table S19. Log-binomial regression models for the percentage of annoyed people (a lot, quite a bit, somewhat) due to airplane noise as a function of modelled noise levels of the total sound environment in LA_{eq24h}.

Variable	PPR	SE	*p*-Value	(95% CI)
LA_{eq24h}	0.989	0.014	0.414	(0.961, 1.016)
Age				
18–29	1			
30–39	1.816	0.513	0.035	(1.044, 3.159)
40–49	2.384	0.626	0.001	(1.424, 3.989)
50–59	2.224	0.587	0.002	(1.325, 3.732)
60–69	1.462	0.401	0.166	(0.854, 2.503)
70–79	1.592	0.460	0.107	(0.904, 2.804)
≥80	1.037	0.363	0.918	(0.521, 2.06)
Education				
No diploma	1			
Highschool	1.294	0.364	0.359	(0.746, 2.246)
Cegep	1.782	0.487	0.035	(1.043, 3.044)
University degree	2.119	0.559	0.004	(1.264, 3.555)
Sex				
Men	1			
Women	1.095	0.109	0.361	(0.901, 1.33)
Constant	0.079	0.071	0.005	(0.013, 0.468)

Table S20. Log-binomial regression models for the percentage of highly annoyed people (a lot, quite a bit) due to the total environment (*i.e.*, any outdoor noise source) as a function of modelled noise levels of the total sound environment in L_{den}.

Variable	PPR	SE	*p*-Value	(95% CI)
L_{den}	1.042	0.010	0.000	(1.024, 1.061)
Age				
18–29	1			
30–39	1.234	0.233	0.267	(0.852, 1.787)
40–49	1.525	0.272	0.018	(1.076, 2.162)
50–59	1.499	0.262	0.021	(1.063, 2.112)
60–69	1.217	0.224	0.285	(0.849, 1.746)
70–79	1.059	0.215	0.776	(0.712, 1.576)
≥80	1.201	0.287	0.444	(0.752, 1.917)
Education				
No diploma	1			
Highschool	1.000	0.165	0.999	(0.724, 1.382)
Cegep	1.281	0.201	0.114	(0.942, 1.742)
University degree	1.130	0.172	0.422	(0.838, 1.523)
Sex				
Men	1			
Women	1.189	0.088	0.019	(1.029, 1.374)
Constant	0.010	0.006	0.000	(0.003, 0.032)

Table S21. Log-binomial regression models for the percentage of annoyed people (a lot, quite a bit, somewhat) due to the total environment (*i.e.*, any outdoor noise source) as a function of modelled noise levels of the total sound environment in L_{den}.

Variable	PPR	SE	*p*-Value	(95% CI)
L_{den}	1.032	0.005	0.000	(1.022, 1.043)
Age				
18–29	1			
30–39	1.181	0.129	0.129	(0.953, 1.463)
40–49	1.216	0.128	0.064	(0.989, 1.496)
50–59	1.175	0.123	0.125	(0.956, 1.443)
60–69	0.989	0.110	0.924	(0.796, 1.23)
70–79	1.002	0.123	0.985	(0.787, 1.276)
≥80	0.887	0.143	0.454	(0.647, 1.215)
Education				
No diploma	1			
Highschool	1.143	0.140	0.276	(0.898, 1.454)
Cegep	1.378	0.160	0.006	(1.097, 1.731)
University degree	1.456	0.164	0.001	(1.167, 1.816)
Sex				
Men	1			
Women	1.062	0.050	0.199	(0.969, 1.164)
Constant	0.037	0.013	0.000	(0.018, 0.075)

Table S22. Log-binomial regression models for the percentage of highly annoyed people (a lot, quite a bit) due to the total environment (*i.e.*, any outdoor noise source) as a function of modelled noise levels of the total sound environment in LA_{eq24h}.

Variable	PPR	SE	*p*-Value	(95% CI)
LA_{eq24h}	1.041	0.010	0.000	(1.022, 1.06)
Age				
18–29	1			
30–39	1.242	0.234	0.250	(0.858, 1.798)
40–49	1.521	0.271	0.019	(1.073, 2.156)
50–59	1.504	0.263	0.020	(1.068, 2.118)
60–69	1.221	0.224	0.276	(0.852, 1.751)
70–79	1.054	0.214	0.796	(0.708, 1.569)
≥80	1.207	0.288	0.432	(0.756, 1.927)
Education				
No diploma	1			
Highschool	0.986	0.164	0.933	(0.712, 1.365)
Cegep	1.268	0.200	0.132	(0.931, 1.726)
University degree	1.128	0.173	0.432	(0.835, 1.523)
Sex				
Men	1			
Women	1.189	0.088	0.019	(1.03, 1.374)
Constant	0.013	0.007	0.000	(0.004, 0.039)

Table S23. Log-binomial regression models for the percentage of annoyed people (a lot, quite a bit, somewhat) due to the total environment (*i.e.*, any outdoor noise source) as a function of modelled noise levels of the total sound environment in LA_{eq24h}.

Variable	PPR	SE	*p*-Value	(95% CI)
LA_{eq24h}	1.030	0.006	0.000	(1.02, 1.041)
Age				
18–29	1			
30–39	1.184	0.130	0.123	(0.956, 1.467)
40–49	1.212	0.128	0.069	(0.985, 1.491)
50–59	1.176	0.124	0.123	(0.957, 1.445)
60–69	0.992	0.110	0.943	(0.798, 1.233)
70–79	1.002	0.124	0.987	(0.786, 1.277)
≥80	0.892	0.144	0.478	(0.651, 1.223)
Education				
No diploma	1			
Highschool	1.134	0.140	0.307	(0.891, 1.443)
Cegep	1.373	0.160	0.007	(1.093, 1.725)
University degree	1.457	0.165	0.001	(1.167, 1.819)
Sex				
Men	1			
Women	1.062	0.050	0.202	(0.968, 1.164)
Constant	0.048	0.017	0.000	(0.024, 0.095)

Table S24. Estimated marginal proportions of highly annoyed (a lot, quite a bit) persons in the total study population and stratified by noise sensitivity (with 95% CI) from total environmental noise (includes noise from transport, neighborhood, industrial and commercial sources, schools, parks, animals and birds) as a function of L_{den}, adjusted for age, education and sex.

L_{den} (dBA)	Total Sample		Sensitive to Noise (a Lot, Quite a Bit)		Not Sensitive to Noise (Not at All, a Little, Somewhat)	
	%	95% CI	%	95% CI	%	95% CI
45	10.5	(6.7, 14.3)	12.5	(4.7, 20.4)	10.6	(5.9, 15.2)
50	12.9	(9.3, 16.5)	16.3	(8.5, 24.1)	12.6	(8.4, 16.8)
55	15.9	(12.8, 18.9)	21.2	(14.1, 28.3)	15.0	(11.6, 18.5)
60	19.5	(17.3, 21.7)	27.6	(22.1, 33.1)	17.9	(15.5, 20.4)
65	24.0	(22.3, 25.7)	35.9	(31.6, 40.3)	21.4	(19.6, 23.2)
70	29.5	(26.1, 33.0)	46.7	(38.0, 55.5)	25.5	(21.9, 29.1)
75	36.3	(29.3, 43.4)	60.8	(41.4, 80.3)	30.5	(23.2, 37.7)

© 2015 by the authors; licensee MDPI, Basel, Switzerland. This article is an open access article distributed under the terms and conditions of the Creative Commons by Attribution (CC-BY) license (http://creativecommons.org/licenses/by/4.0/).
